# Smooth Muscle Cell Functionality on Collagen Immobilized Polycaprolactone Nanowire Surfaces

**DOI:** 10.3390/jfb5020058

**Published:** 2014-05-08

**Authors:** Victoria Leszczak, Dominique A. Baskett, Ketul C. Popat

**Affiliations:** 1 Department of Mechanical Engineering, Colorado State University, Fort Collins, CO 80523, USA; E-Mail: Victoria.Leszczak@colostate.edu; 2 Department of Biomedical Science, Colorado State University, Fort Collins, CO 80523, USA; E-Mail: dbaskett@rams.colostate.edu; 3 School of Biomedical Engineering, Colorado State University, Fort Collins, CO 80523, USA

**Keywords:** nanowires, smooth muscle cells, contractile phenotype, synthetic phenotype, collagen I

## Abstract

Inhibition of smooth muscle cell (SMC) proliferation and preservation of a differentiated state are important aspects in the management, avoidance and progression of vascular diseases. An understanding of the interaction between SMCs and the biomaterial involved is essential for a successful implant. In this study, we have developed collagen immobilized nanostructured surfaces with controlled arrays of high aspect ratio nanowires for the growth and maintenance of human aortic SMCs. The nanowire surfaces were fabricated from polycaprolactone and were immobilized with collagen. The objective of this study is to reveal how SMCs interact with collagen immobilized nanostructures. The results indicate significantly higher cellular adhesion on nanostructured and collagen immobilized surfaces; however, SMCs on nanostructured surfaces exhibit a more elongated phenotype. The reduction of MTT was significantly lower on nanowire (NW) and collagen immobilized NW (colNW) surfaces, suggesting that SMCs on nanostructured surfaces may be differentiated and slowly dividing. Scanning electron microscopy results reveal that SMCs on nanostructured surfaces are more elongated and that cells are interacting with the nano-features on the surface. After providing differentiation cues, heavy chain myosin and calponin, specific to a contractile SMC phenotype, are upregulated on collagen immobilized surfaces. These results suggest that nanotopography affects cell adhesion, proliferation, as well as cell elongation, while collagen immobilized surfaces greatly affect cell differentiation.

## 1. Introduction

The vascular smooth muscle cell’s (SMC) main function is contraction and adjustment of blood vessel diameter, blood pressure and blood flow distribution [1]. SMCs have the capacity for contraction, migration, proliferation, synthesis of extracellular matrix (ECM) components and the secretion of growth factors and cytokines [2]. This allows SMCs to regulate lumen diameter both transiently and chronically [3]. SMCs are key players in the development of vascular disease, due to their plasticity or ability to change phenotype and behavior according to varying environmental conditions [4]. The standard treatment for vascular disease is coronary angioplasty, which leads to the disorder of the endothelial layer. This leaves a highly prothrombotic surface exposed to the blood stream and promotes SMC dedifferentiation followed by proliferation [5,6]. Restoration of an endothelium represents a crucial process in re-establishing an intact vessel surface, but in order to do this, SMC proliferation must be controlled. Enhanced SMC proliferation often leads to restenosis, the reocclusion of the blood vessel. This can be caused by SMC migration, proliferation and neointimal thickening and further limits the success of balloon angioplasty and stent implantation [7].

SMCs exhibit two well-known phenotypes; contractile and synthetic [8]. Contractile SMCs are elongated and spindle shaped, whereas synthetic SMCs are less elongated and have an epithelioid morphology. Synthetic SMCs contain organelles involved in protein synthesis, whereas in contractile SMCs, these organelles are replaced with contractile filaments. Further, synthetic and contractile SMCs exhibit different proliferation rates. Synthetic SMCs proliferate at higher rates compared to contractile SMCs. Contractile SMCs in adult blood vessels proliferate at an exceptionally low rate, demonstrate low synthetic activity and express a unique selection of contractile proteins and signaling molecules required for contractile function [9]. SMCs within adult blood vessels are incredibly plastic and can easily change phenotype, a process also known as phenotypic switching. This occurs in response to vascular injury, allowing them to proliferate at high rates [10,11]. SMC phenotypic switching is characterized by a decrease in the expression of SMC-specific differentiation proteins and increased SMC proliferation, migration and synthesis of ECM components required for the repair of vasculature. An unfortunate consequence of SMC plasticity is that environmental cues and signals can promote SMC phenotypic switching and stimulate the development and/or progression of vascular disease [2]. There is evidence that phenotypic switching of SMCs plays a significant role in a repertoire of diseases in humans, such as atherosclerosis, asthma, hypertension and cancer [4]. A decrease in proliferation, however, is not sufficient to promote SMC differentiation [2].

Biomaterial-cell interactions are fundamental in various biological events and determine the longevity and functionality of implanted devices. It is well known that cardiovascular biomaterial implants, such as stents, perturb the endothelium layer, promoting SMC migration and proliferation, ultimately leading to restenosis and the failure of the implant [12]. However, this may be avoidable with the design of a biomaterial surface capable of influencing cellular interactions. Enhancement of cell-biomaterial interactions can be altered by immobilizing surfaces with extracellular matrix (ECM) molecules, such as fibronectin, laminin, collagen or vitronectin [13]. By modifying biomaterial surfaces with these ECM components, cell-binding sites are introduced that may promote cell functionality. One such ECM molecule is collagen 1. It is the main component in the ECM of blood vessels, as well as other tissues in the body and has been used extensively to promote smooth muscle cell adhesion on biomaterial surfaces [14,15]. Further, it is also known to promote the contractile phenotype in SMCs [16]. Another popular approach for controlling cell functionality on implant surfaces is the introduction of surface topographies at a nanometer or micrometer scale. Due to the existence of functional nanoscale structures within native tissue, nanostructured surfaces have attracted attention. Nanoscale features can influence cellular responses, ranging from initial attachment and migration to differentiation and synthesis of new ECM. Studies have shown that proliferation of the SMCs is significantly reduced on nanopatterned surfaces, while these surfaces also promote the alignment of cells [17,18]. Topographical features that mimic the natural extracellular matrix have also been shown to encourage SMC attachment and bioactivity [19,20], while limiting proliferation [17].

In this study, we have used polycaprolactone, since it has exceptional properties for implantation, such as outstanding mechanical strength and a low degradation rate in physiological conditions. It can also easily be processed to have nanoscaled features. Further, its degradation products are easily bioresorbed or removed naturally in metabolic pathways, such as the citric acid cycle. Polycaprolactone has therefore received a great deal of attention for use as an implantable biomaterial for many tissue engineering applications, including cardiovascular applications [21,22,23].

In this work, we present the immobilization of collagen I onto polycaprolactone nanowire surfaces. Polycaprolactone nanowire surfaces were fabricated by a solvent-free template synthesis technique developed for fabricating controlled arrays of a high aspect ratio; substrate-bound nanowires from polycaprolactone. This nanotopography was chosen, since it has shown promising hemocompatible properties [24] and transcellular growth capabilities, specifically neurons [25] and mesenchymal stem cells [26]. However, not much is known about how SMCs will interact with this surface. It is envisioned that the incorporation of collagen on nanowires may facilitate the adherence and differentiation of SMCs. The objective of this work is to investigate how SMCs interact with nanowire topography to see if these surfaces have potential for cardiovascular implants. Understanding how to anchor smooth muscle cells and the interaction of the cell surface receptors with the ECM components will provide a foundation for developing functional vascular biomaterials that inhibit unwarranted vascular SMC proliferation and preserve a differentiated state of SMCs to manage and avoid vascular disease conditions.

The notation for the rest of the manuscript is as follows: control polycaprolactone (PCL), control + collagen (colPCL), nanowire (NW) and nanowire + collagen (colNW).

## 2. Results and Discussion

Understanding how SMCs respond to biomaterial surfaces, as well as how cell surface receptors interact with the ECM components will provide a foundation for developing functional biomaterials designed to promote a contractile SMC phenotype. In general, SMC phenotypic switching is characterized by markedly reduced expression of SMC-selective differentiation marker genes and increased SMC proliferation, migration and synthesis of extracellular matrix components required for vascular repair. This can often lead to restenosis and can progress the development of vascular disease. In this study, we explore the effect of the combination of both surface nanoarchitecture and cell binding motifs on SMC adhesion, viability, proliferation and differentiation.

### 2.1. Characterization of Surfaces

**Figure 1:**
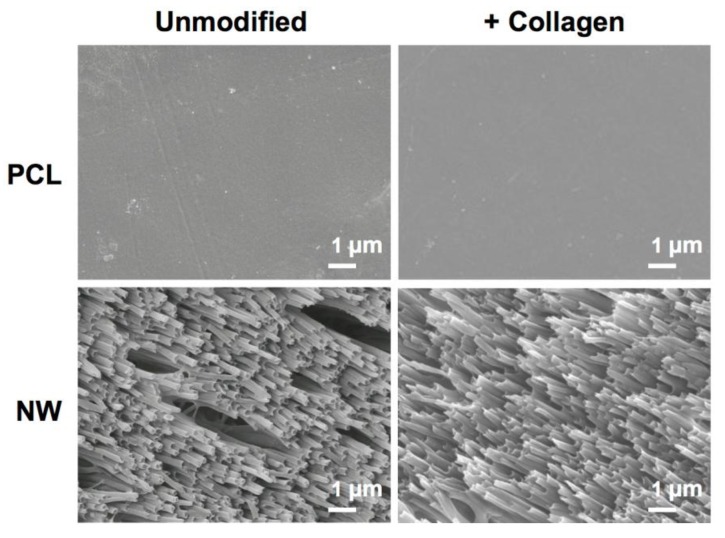
Representative SEM images of control polycaprolactone (PCL) and nanowire (NW) surfaces before and after collagen immobilization.

The surface architecture of the different surfaces before and after the collagen immobilization process was characterized using SEM. Results reveal that the surface architecture remains consistent before and after collagen immobilization (Figure 1).

**Figure 2:**
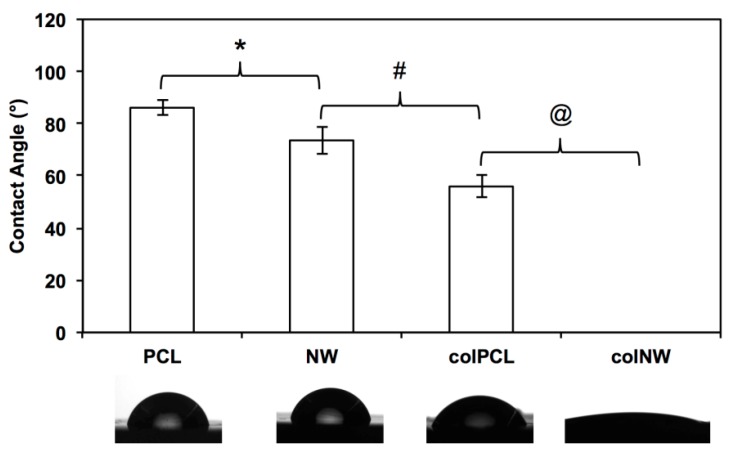
Contact angle measurements of PCL, NW, colPCL (col, collagen) and colNW surfaces. Experiments were replicated on at least five different samples (*n*_min_ = 5). Statistical significance was calculated using a one-way ANOVA with Tukey’s *post hoc* test. Results indicate significant differences between contact angles on all surfaces (*, #, @, *p* < 0.05). The contact angle on colNW surfaces could not be obtained.

Surface wettability was characterized by measuring the water contact angle using a goniometer (Model 250 Standard Goniometer, ramé-hart, Succasunna, NJ, USA). Results show significantly different contact angles between all four different surfaces (PCL > NW > colPCL > colNW) (Figure 2). The contact angle is the angle where a liquid/vapor interface meets a solid surface and is also dependent on the surface area. Further, a lower contact angle is associated with higher surface energies. NW and colNW surfaces have an increased amount of surface area compared to PCL and colPCL surfaces, indicating why they have lower contact angles. A decrease in contact angle measurements occurs once collagen is immobilized on to the surfaces. The increase in polarity of the surfaces caused by the polar groups of collagen indicates why the surface energy increases on collagen immobilized surfaces.

**Figure 3:**
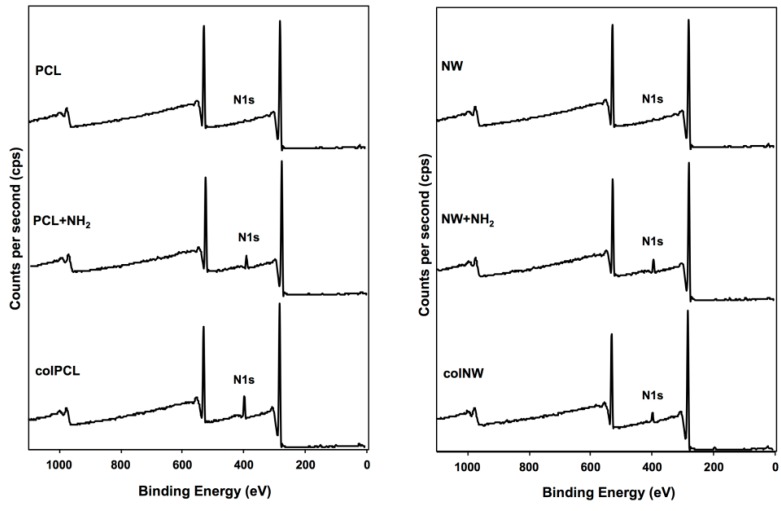
Survey XPS scans of PCL, NW, colPCL and colNW surfaces. Results show an increase in the N1s peak throughout the collagen immobilization process.

The surface composition was analyzed using X-ray photoelectron spectroscopy (XPS). Survey scans were taken after each step in the immobilization process in order to determine the overall composition of the different surfaces. Results indicate an increase in the N1s peak after the aminolysis step. A further increase in the N1s peak was observed after collagen immobilization (Figure 3).

### 2.2. Adhesion and Proliferation of SMCs

**Figure 4:**
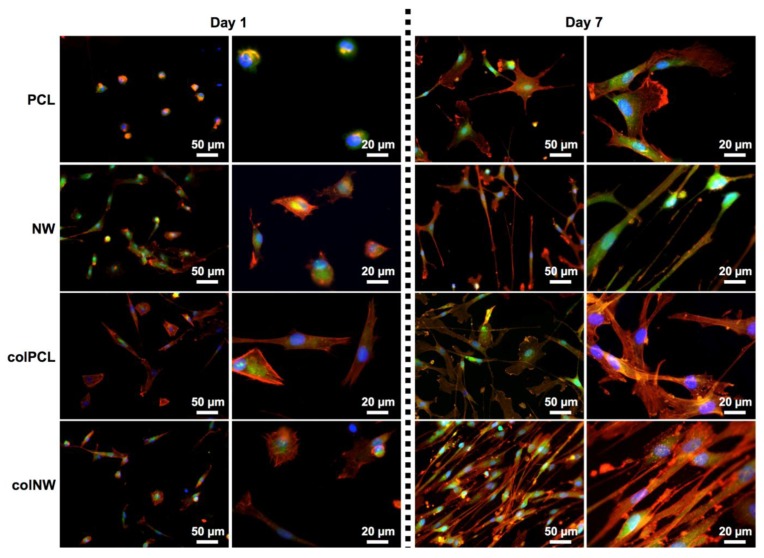
Representative fluorescence microscopy images of SMCs stained with 5-chloromethylfluorescein diacetate (CMFDA) (green), rhodamine phalloidin (red) and DAPI (blue) on the PCL, NW, colPCL and colNW surfaces. Experiments were replicated on at least three different samples with at least three different cell populations (*n*_min_ = 9).

SMC adhesion and proliferation was evaluated after one and seven days of culture by using 5-chloromethylfluorescein diacetate (CMFDA) live stain (Invitrogen, Carlsbad, NM, USA), rhodamine phalloidin (Cytoskeleton) and 4',6-diamidino-2-phenylindole dihydrochloride (DAPI) (Invitrogen) nucleus stain, followed by imaging with a fluorescence microscope (Figure 4). At Day 1, it is evident that cells adhered onto the NW, colPCL and colNW surfaces have an elongated phenotype compared to PCL surfaces. SMCs on NW, colPCL and colNW also exhibit multiple cellular extensions interacting with the surfaces, as well as with surrounding cells. After seven days of culture, there are more cells covering and interacting on both the colPCL and colNW surfaces, as compared to the PCL and NW surfaces. It is important to note that the cells on the nanostructured surfaces (NW and colNW) seem to be aligning and more spindle-shaped compared to the flat surfaces (PCL and colPCL).

**Figure 5:**
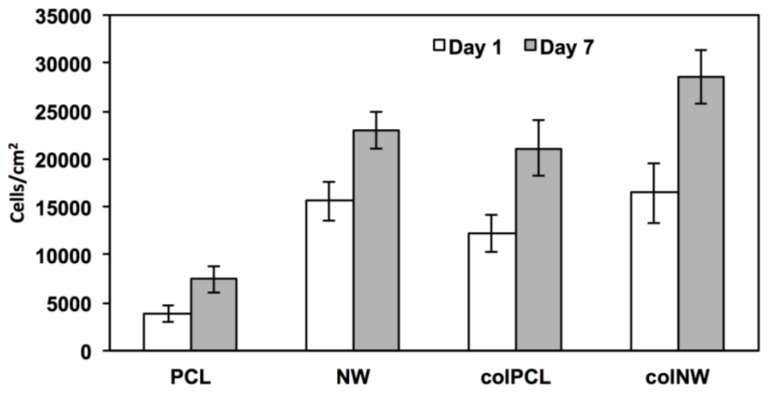
Cell counts on different surfaces after one and seven days of culture. Cell nuclei stained with DAPI were quantified using ImageJ software. Experiments were replicated on at least three different samples with at least three different cell populations (*n*_min_ = 9). Statistical significance was calculated using a one-way ANOVA with Tukey’s *post hoc* test. After one day in culture, the cellular adhesion of SMCs on PCL surfaces is significantly lower than the adhesion on NW and colNW surfaces, whereas there is no significant difference between cellular adhesion on colPCL, NW and colNW surfaces. After seven days in culture, the colPCL, NW and colNW surfaces exhibit significantly higher cellular adhesion than PCL surfaces, whereas, there is no significant difference between cellular adhesion on the colPCL, NW and colNW surfaces. Error bars represent the standard error.

SMC adhesion was quantified by counting the number of DAPI-stained nuclei on fluorescence microscopy images using ImageJ software. The results indicate that after one day in culture, SMC adhesion on PCL surfaces is statistically similar to that on colPCL surfaces; however, adhesion on PCL surfaces is significantly lower than that on NW and colNW surfaces (Figure 5). After seven days of culture, the NW, colPCL and colNW surfaces exhibit higher cellular adhesion, as compared to PCL surfaces. There is also a significant increase in cellular adhesion on colNW surfaces from Day 1 to 7. This may be due to the enhanced energy of these surfaces compared to the PCL surface. Higher surface energy is known to promote cell adhesion. Further, collagen immobilized onto these surfaces presents more cell binding motifs to which cells can adhere. In natural tissue, cells are surrounded with ECM; therefore, cells will interact with a biomaterial in a comparable way if it contains similar binding sites to that of the natural ECM [27]. Collagen has been known to increase cellular anchorage to substrates via the ß_1_ integrin family of extracellular matrix receptors [28,29]. Cellular adhesion plays a large role in cellular communication and regulation, indicating that collagen immobilized surfaces may serve as an excellent substrate for the adhesion of SMCs. The nanostructured and collagen immobilized surfaces promote initial cellular adhesion, and hence, there are more cells on these surfaces. By introducing nanoarchitecture, as well as a cell binding motif, it is apparent that more cells are adhering, indicating that mimicking the natural-like hierarchy of tissue and providing ECM components are important in anchoring SMCs.

### 2.3. SMC Elongation on Different Surfaces

**Figure 6:**
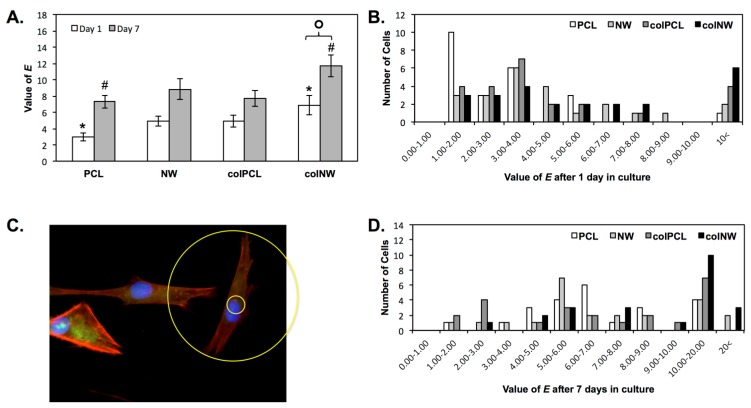
(**A**) Elongation (*E*) approximations of cells on different surfaces after one and seven days of culture. Experiments were replicated on at least three different samples with at least three different cell populations (*n*_min_ = 9). Statistical significance was calculated using a one-way ANOVA with Tukey’s *post hoc* test. Results indicate that colNW surfaces exhibit a more elongated morphology after one and seven days of culture compared to PCL surfaces at the same time points (*, #, *p* < 0.05). SMCs also become significantly more elongated on colNW surfaces after seven days of culture compared to those in culture for one day on colNW surfaces (¢, *p* < 0.05). Error bars represent the standard error; (**B**) The image shows how *E* approximations were calculated (outer diameter/inner diameter); A histogram of *E* approximations of cells on different surfaces after (**C**) one day in culture and (**D**) seven days in culture.

SMCs adhered onto different surfaces after one and seven days of culture with evident boundaries were examined using ImageJ software to acquire an approximation for cellular elongation. Cellular elongation was calculated as the aspect ratio of cellular length to cellular width, outputting an elongation (*E*) parameter. Results indicate that colNW surfaces exhibit a more elongated morphology after one and seven days of culture compared to PCL surfaces at the same time points (Figure 6A). SMCs also exhibited significantly more elongation on colNW surfaces after seven days of culture as compared to those after one day of culture. Contractile SMCs are more elongated and spindle-shaped cells, whereas synthetic SMCs have a cobblestone morphology, also known as epithelioid. Thus, a higher *E* value is associated with a contractile phenotype. The large error in the average *E* value is attributed to the vast distribution of cellular elongation on the surfaces. Therefore, a histogram of cellular *E* values on the different surfaces was constructed in order to see the distribution of cellular shapes (Figure 6C,D). After one day in culture, the histogram indicates that the majority of cells on PCL surfaces have an *E* value between one and two. SMCs on NW and colPCL surfaces have a large distribution of cell shapes with peaks being between *E* values of three and four. Cells on colNW surfaces, which contain both nanoarchitecture, as well as cell binding motifs, have the largest number of elongated cells (*E* values greater than 10). Further, after seven days of culture, the NW and colNW surfaces are the only surfaces that adhere SMCs with an *E* value greater than 20.

### 2.4. Viability of SMCs on Surfaces

**Figure 7:**
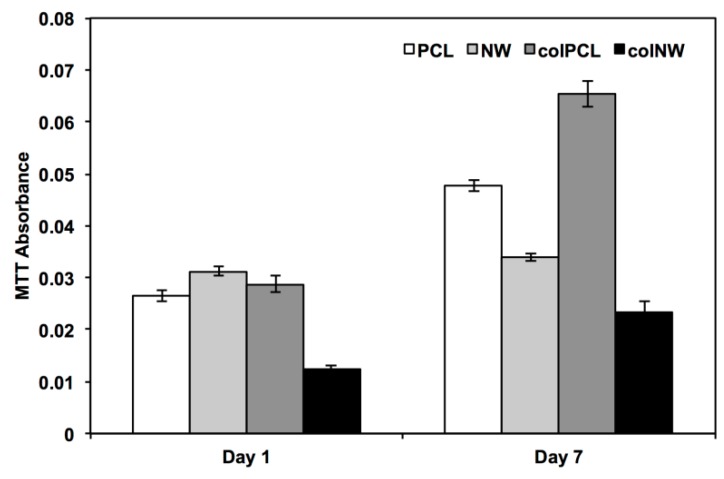
Cell viability measured using the MTT assay for SMCs on different surfaces. Experiments were replicated on at least three different samples with at least three different cell populations (*n*_min_ = 9). Statistical significance was calculated using a one-way ANOVA with Tukey’s *post hoc* test. Results indicate after one day in culture that SMCs on the colNW surfaces have significantly lower MTT reduction than the PCL, NW and colPCL surfaces, whereas, there is no significant difference between the PCL, NW and colPCL surfaces. After seven days in culture, SMCs have a significantly higher MTT reduction on colPCL than the PCL, NW and colNW surfaces. Further, there were significant differences between PCL, NW and colNW surfaces (PCL > NW > colNW). Error bars represent the standard error.

The cell viability was characterized using a commercially available methylthiazol tetrazolium (MTT) assay kit (Sigma, St. Louis, MO, USA) after Days 1 and 7 in culture. This assay is dependent on NAD(P)H-dependent oxidoreductase enzymes located in the cytosolic compartment of cells, which reduce the tetrazolium dye [30,31]. Therefore, this assay can be used to measure the loss of viable cells or the cytostatic activity of cells. Results indicate after one day in culture that SMCs on the colNW surfaces have significantly lower MTT reduction rates than the PCL, NW and colPCL surfaces (Figure 7). After seven days in culture, SMCs have significantly higher MTT reduction rates on colPCL > (PCL > NW > colNW). Studies have shown that proliferation of smooth muscle cells is significantly reduced on nanopatterned surfaces [21]. Rapidly dividing cells demonstrate high rates of MTT reduction, while differentiated cells exhibit low rates of MTT reduction. This can be seen in the lower values of MTT reduction of SMCs on NW and colNW surfaces compared to both PCL and colPCL surfaces. Despite a significantly higher number of cells on the colNW surfaces after Day 7 of culture as compared to the PCL, NW and colPCL surfaces, the cells on the colNW surfaces are significantly more elongated than the other surfaces, indicating that they may have a differentiated phenotype. Contractile SMCs in adult blood vessels proliferate at an extremely low rate and exhibit low synthetic activity, so despite the fact that there are more cells on the colNW surfaces, these cells may be in a quiescent state.

### 2.5. SMC Morphology on Different Surfaces

**Figure 8:**
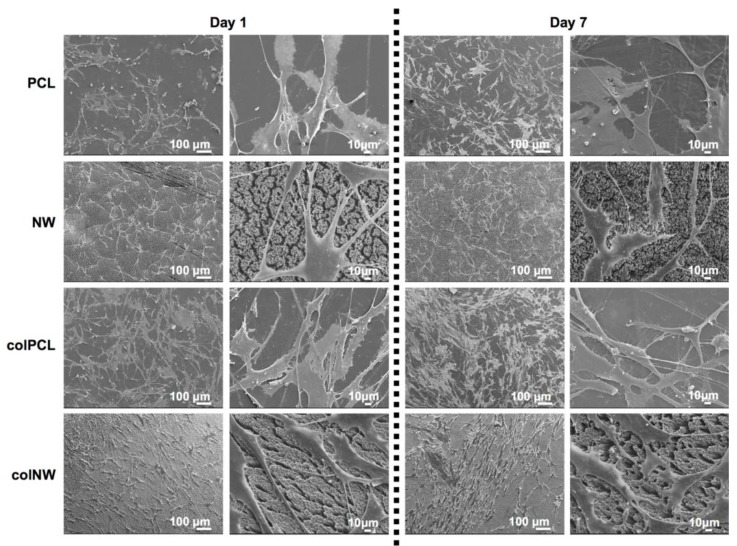
Representative SEM images of SMCs after one and seven days of culture on different surfaces. Note: the surfaces were coated with a 10-nm layer of gold and imaged at 7 keV. Experiments were replicated on at least three different samples with at least three different cell populations (*n*_min_ = 9).

SMC morphology was investigated using SEM imaging to visualize the cellular interaction with the surface nanoarchitecture. The nanoscale surface topography of materials has been shown to be significant in interactions with biological systems, such as proteins and cells [32]. Results indicate that there is a clear interaction between SMC’s filopodia and the nano-features present on both NW and colNW surfaces, as well as with the collagen immobilized surfaces (colPCL and colNW) (Figure 8). After one day in culture, it is evident that there are SMCs present on the PCL and colPCL surfaces with a round morphology, as opposed to SMCs adhered on to the NW and colNW surfaces. Further, confirming fluorescent imaging results, it is apparent that SMCs on the NW and colNW surfaces seem to be more elongated than those on the PCL and colPCL surfaces after one and seven days of culture. After seven days in culture, SMCs on the PCL and colPCL surfaces are no longer round, but are still not nearly as elongated as SMCs on the NW and colNW surfaces. SMCs do not seem to interact as well with PCL surfaces when compared to the NW, colPCL and colNW surfaces, as is evident by the clear lack of filopodia. This may be due to the lack of nano-architecture and cell binding motifs.

### 2.6. Differentiation of SMCs on Different Surfaces

Expression of SMC contractile proteins is essential to healthy vasculature, as cardiovascular diseases are characterized by the transition of the SMC phenotype from contractile to synthetic. During the differentiation of SMCs, caldesmon, smooth muscle myosin heavy chain, calponin, SM22, α- and β-tropomyosins and αl integrin genes are transcriptionally regulated. However, in differentiated SMCs, the transcription of these genes is upregulated in differentiated SMCs and downregulated in dedifferentiated SMCs [33]. Downregulation of SMC lineage markers has been shown to be associated with calcification [34]. Therefore, the differentiation of SMCs on surfaces was investigated by detecting CAL and MYH expression, both through blotting techniques after seven, 14 and 21 days in culture and with immunofluorescence after 21 days in culture and western blotting. CAL and MYH are specific to smooth muscle cells and are expressed only when smooth muscles cells differentiate into a mature phenotype. CAL is a calcium binding protein, located in the thin filaments of smooth muscle. It is present at a stoichiometry of 1 mol calponin/7 mol actin [35]. MYH is involved in contraction and is a highly specific marker for the SMC lineage. The expression of MYH has never been found in cells other than SMCs *in vivo* and MYH is the only marker protein that is also SMC-specific during embryogenesis [36].

**Figure 9:**
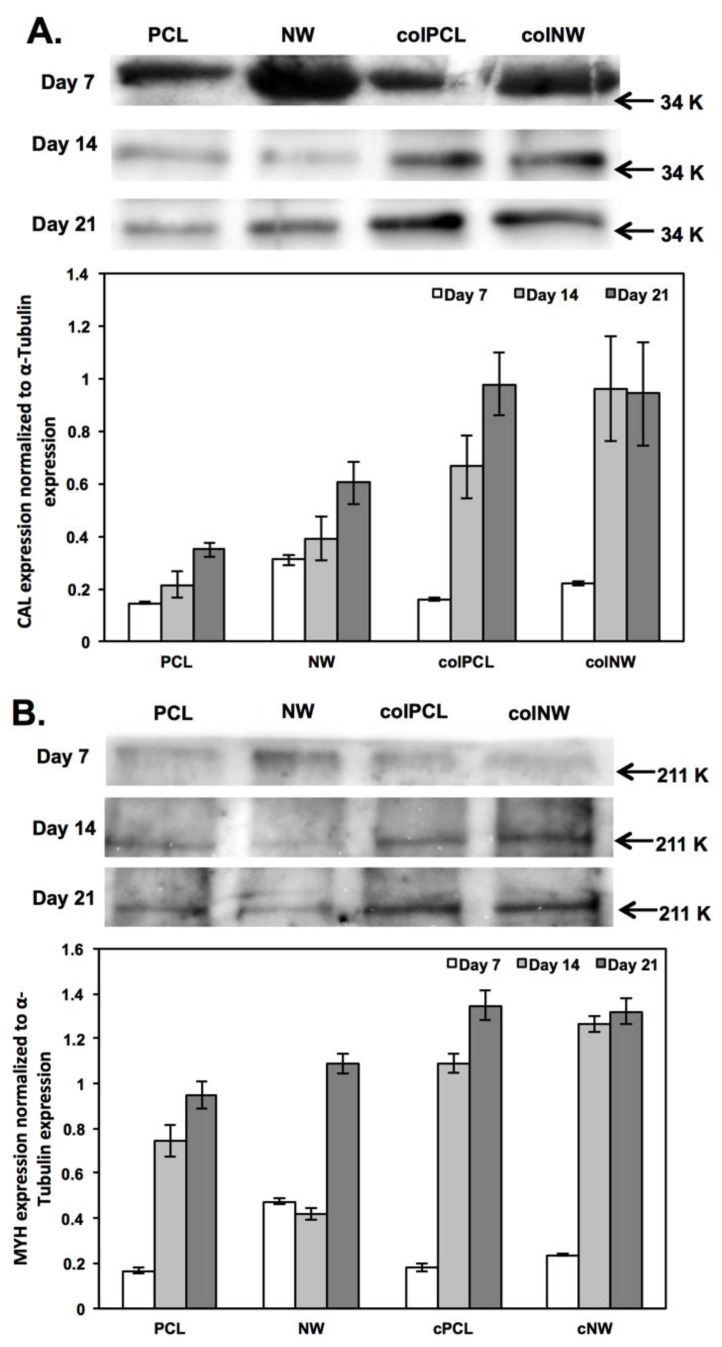
Western blot analysis of the expression of (**A**) CAL and (**B**) MYH on different surfaces after seven, 14 and 21 days in culture. Experiments were replicated with western blots with at least three different cell populations (*n*_min_ = 9). Statistical significance was calculated using a one-way ANOVA with Tukey’s *post hoc* test. After seven days in culture, NW and colNW express significantly more amounts of CAL and MYH compared to the PCL and colPCL surfaces, while the NW surfaces express CAL and MYH significantly more than colNW. After 14 days in culture, CAL expression is significantly higher on the colNW surfaces compared to the PCL and NW surfaces. After 14 days in culture, both collagen immobilized surfaces (colPCL and colNW) express significantly more MYH than the PCL and NW surfaces, while the PCL surfaces express significantly more MYH than the NW surfaces. After 21 days in culture, CAL and MYH expression is elevated significantly on the colPCL and colNW surfaces compared to the PCL and NW surfaces.

A western blotting technique was used to partially quantify the presence of MYH and CAL on the surfaces. The MYH and CAL expressions were normalized to α-tubulin expression. After seven days in culture, differentiation was investigated without supplying the cells with differentiation media. Results reveal that the cells are beginning to differentiate on all surfaces. CAL (Figure 9A) and MYH (Figure 9B) expression is significantly higher on NW surfaces compared to PCL, colPCL and colNW surfaces, while colNW surfaces express significantly higher amounts of CAL and MYH compared to PCL and colPCL surfaces. This indicates that cells are significantly more differentiated on nanostructured surfaces at Day 7, explaining why MTT results were significantly lower on these surfaces. It is important to note that the bands for Day 7 are larger than for Days 14 and 21, because more protein lysate was loaded after seven days in culture, compared to after 14 and 21 days in culture. However, all bands were normalized to α-tubulin expression to account for this. After 14 days in culture (seven days after providing differentiation media), CAL expression is significantly higher on the colNW surfaces compared to the PCL and NW surfaces. Both collagen immobilized surfaces (colPCL and colNW) express significantly more MYH than the PCL and NW surfaces, while the PCL surfaces express significantly more MYH than NW surfaces. After 21 days in culture, CAL and MYH expression is elevated significantly on the colPCL and colNW surfaces compared to the PCL and NW surfaces. These results are in agreement with a study that found that collagen type I, type IV and laminin promoted the contractile/differentiated phenotype in SMCs [16].

**Figure 10:**
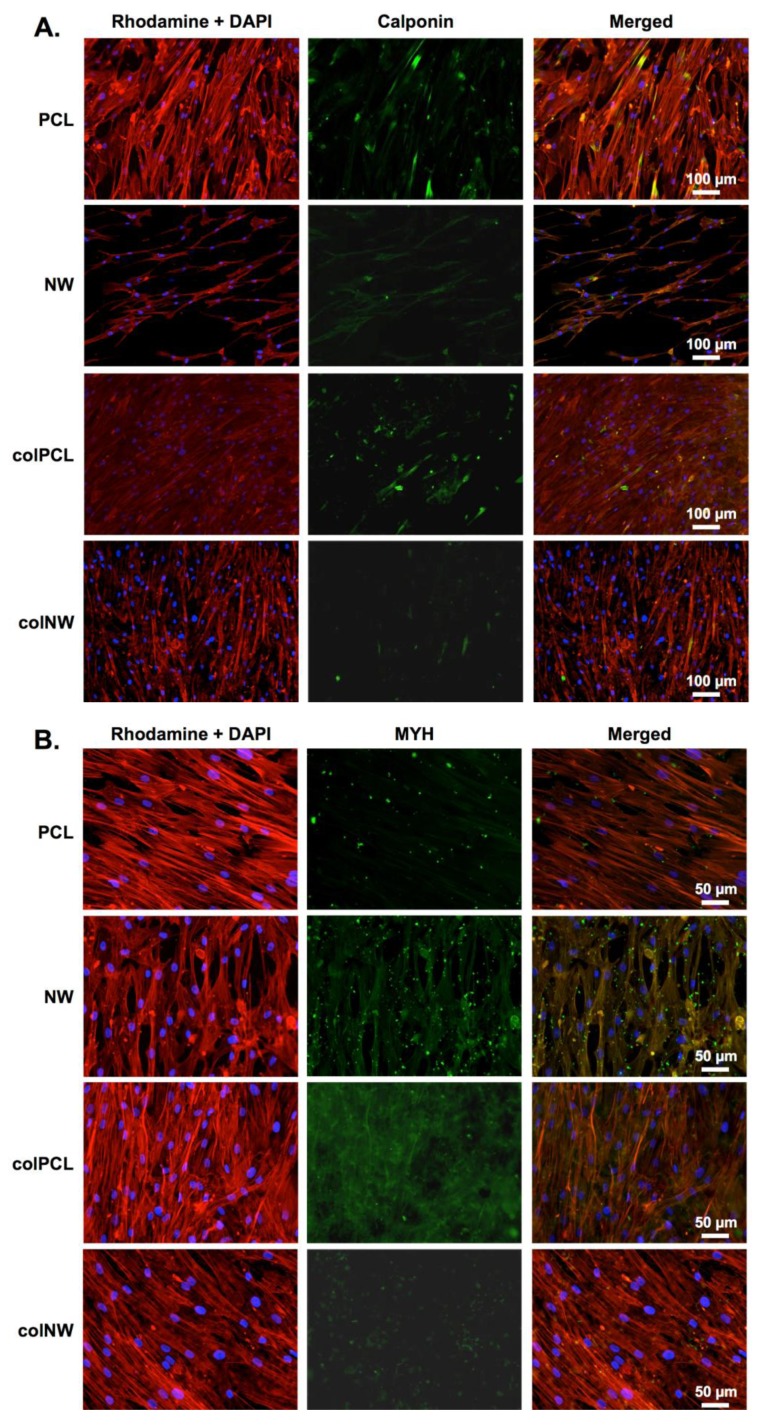
Representative fluorescence microscopy images of SMCs immunostained with (**A**) CAL and (**B**) MYH. Experiments were replicated on at least three different samples with at least three different cell populations (*n*_min_ = 9).

Immunofluorescence was done after 21 days in culture. The results of immunofluorescence indicate that cells on all surfaces are expressing both CAL (Figure 10A) and MYH (Figure 10B). It is important to note that even with differentiation media, the SMCs are proliferating on all surfaces, but more so on the PCL surfaces. Further, SMCs on all surfaces exhibit an elongated spindle-shaped morphology.

## 3. Experimental Section

### 3.1. Fabrication of PCL Nanowire Surfaces

Control (smooth PCL) surfaces were fabricated by sintering PCL pellets (MW = 80,000, Sigma) on a glass plate in a 10-mm Teflon washer (Ace Hardware, Fort Collins, CO, USA). The resulting discs were then air-cooled before using a biopsy punch to ensure a common diameter between all surfaces (10 mm).

PCL nanowire surfaces were fabricated utilizing a solvent-free nanotemplating technique with 20-nm diameter nanoporous aluminum oxide membranes [37]. PCL discs were placed on the membrane surface and placed in an oven at 115 °C for 3–5 min, allowing the nanowires to gravimetrically extrude through the membrane. The aluminum oxide membranes were then dissolved in 1 M NaOH for 75 min. Surfaces were then washed in DI water (3×), dried and stored in a desiccator until their use was required.

Prior to any further use, all surfaces were sterilized in 70% ethanol for 30 min, followed by washing with PBS (2×). Surfaces were then air dried and further sterilized by UV exposure for 30 min.

### 3.2. Immobilization of Collagen on PCL and NW Surfaces

Surfaces fabricated as previously described in Section 2.1 were immobilized with collagen in three steps. First, the surfaces underwent aminolysis by incubation in 1,6-hexanediamine/2-propanol (6% w/V) for 10 min at 37 °C followed by rinsing with DI water (3×) to remove free 1,6-hexanediamine. Second, the surfaces were incubated in a glutaraldehyde (1 wt%) solution at 2–4 °C for 24 h and then rinsed with DI water (3×) to remove free glutaraldehyde. Thirdly, the surfaces were incubated in collagen (1% w/V) for 24 h at 2–4 °C. After the incubation, surfaces were rinsed with 0.1 M acetic acid solution, followed by DI water to remove ungrafted collagen.

### 3.3. Characterization of colPCL and colNW Surfaces

The surface architecture of the different surfaces before and after the collagen immobilization process was characterized using scanning electron microscopy. Prior to imaging, the surfaces were coated with a 10-nm layer of gold and imaged at 5–7 kV. Surface morphology was investigated to ensure a stable architecture throughout the collagen immobilization process.

Surface wettability was characterized by measuring the water contact angle using a goniometer (Model 250 Standard Goniometer, ramé-hart, Succasunna, NJ, USA). A droplet of DI water, approximately 1 µL in volume, was formed on the tip of the syringe, and the stage was moved upward so that the droplet contacted and detached onto the surface. The water droplet image on the surface was captured within 5 s after the contact by a camera leveled with the surface. Images were then analyzed with the accompanying DROPimage advanced software to measure the contact angles.

The surface composition was analyzed using X-ray photospectroscopy (XPS). Survey scans were taken after each step in the immobilization process, in order to determine the overall composition of the different surfaces.

### 3.4. Smooth Muscle Cell Culture

Human aortic SMCs (Life Technologies, Carlsbad, NM, USA) were suspended in MCDB 131 media (Life Technologies) enhanced with SMC growth supplement (supplemented with 2 mmol/L glutamine, 100 μg/mL penicillin, and 100 μg/mL streptomycin), added to 75-cm^2^ culture flasks and incubated at standard culture conditions. This study was performed using SMCs that were passage 4.

SMCs were cultured on PCL, NW, colPCL and colNW surfaces in a 48-well plate. Prior to seeding, all surfaces were subjected to 30 min of UV exposure and conditioned for 5 min in 400 μL of culture medium. SMCs cells were seeded at a density of 2 × 10^4^ cells/well. The surfaces were incubated in standard culture conditions in 400 μL of cell-rich medium and investigated for adhesion, proliferation and viability after 1 and 7 days culture. After 7 days in culture, media changes were done with MCDB 131 media enhanced with SMC differentiation supplement (supplemented with 2 mmol/L glutamine, 100 μg/mL penicillin and 100 μg/mL streptomycin). SMC differentiation was investigated after 14 and 21 days of culture.

### 3.5. Adhesion and Proliferation of SMCs on Different Surfaces

Cellular adhesion and proliferation was investigated using 5-chloromethylfluorescein diacetate (CMFDA) live cytoplasm stain (Invitrogen), rhodamine phalloidin F-actin cytoskeleton stain (Cytoskeleton) and 4',6-diamidino-2-phenylindole dihydrochloride (DAPI) nucleus stain (Invitrogen) by fluorescence microscope imaging after 1 and 7 days of culture.

Prior to staining, non-adherent cells were removed by aspirating the medium from the surfaces followed by two gentle rinses with PBS. The surfaces were then transferred to a new 48-well plate and incubated with 10 μM of CMFDA solution in PBS for 30 min at 37 °C and 5% CO_2_ followed by another incubation in PBS for another 30 min at 37 °C and 5% CO_2_. Then, 3.7% formaldehyde was added to fix the cells for 15 min at room temperature. This was followed by 2 gentle rinses in PBS prior to incubating the surfaces in 1% Triton-X 100 for 3 min in order to permeabilize the cells. The surfaces were rinsed in PBS (3 × 5 min) and then incubated in 5 μL/mL of rhodamine-phalloidin solution for 30 min. After 25 min, 1 μL/mL of DAPI was added to the rhodamine-phalloidin solution. All the surfaces were rinsed and stored in PBS until being imaged using a Zeiss Axioplan 2 fluorescence microscope (Zeiss, Thornwood, NY, USA). The number of cells adherent on the surfaces was determined from the number of stained nuclei in the DAPI fluorescence images using ImageJ software.

### 3.6. SMC Elongation on Different Surfaces

SMCs adhered onto colPCL and colNW surfaces after 1 and 7 days of culture with evident boundaries were examined using ImageJ software to acquire an approximation for cellular elongation. Cellular elongation was calculated as the aspect ratio of cellular length to cellular width, outputting an elongation (*E*) parameter [38]. Cellular length was defined by the diameter of the smallest circle that encompassed the entire cell, while cellular width was defined as the diameter of the largest circle that would fit entirely within the cell. *E* provides a description for the extent of equimomental ellipse lengthening. Thus, *E* is zero for a circle and one for an ellipse with an axis ratio of 1:2.

### 3.7. Viability of SMCs on Different Surfaces

The cell viability was characterized using a commercially available methylthiazol tetrazolium (MTT) assay kit (Sigma) on days 1 and 7 in culture. Prior to measuring mitochondrial activity, the unadhered SMCs were removed by aspirating the cell-rich media from the surfaces followed by two gentle rinses with PBS. Surfaces were transferred to a new 24-well plate and incubated in 10% MTT solution in PBS for 3.5 h at 37 °C and 5% CO_2_. The subsequent formazan crystals were dissolved by adding a 10% Triton-X in MTT solvent mixture in equal amounts to the MTT solution in PBS. The absorbance of the solution was measured at a wavelength of 690 nm using a plate reader (BMG Labtech, Cary, NC, USA). The mitochondric activity of the cells on different surfaces correlate to the resulting absorbance values.

### 3.8. Morphology of SMCs on Different Surfaces

SMC morphology was investigated using SEM imaging to visualize the cellular interaction with the nanoarchitecture. The unadhered cells were removed by aspirating media from the surfaces followed by two gentle rinses with PBS. The surfaces were then transferred to a clean petri-dish, where the cells were fixed and dehydrated on the surface. The cells were fixed by incubating the surfaces in a solution of primary fixative [3% glutaraldehyde (Sigma), 0.1 M sodium cacodylate (Polysciences) and 0.1 M sucrose (Sigma)] for 45 min. Surfaces were then incubated in a solution of secondary fixative (primary fixative without glutaraldehyde) for 10 min. Next, the surfaces were dehydrated by incubation in consecutive solutions of increasing ethanol concentrations (35%, 50%, 70%, 95% and 100%) for 10 min each. Further dehydration of the cells was accomplished by incubating the surfaces in hexamethyldisilazane (HMDS, Sigma) for 10 min. Surfaces were then air dried and stored in a desiccator until imaging by SEM. The surfaces were coated with a 10-nm layer of gold and imaged at 5–7 kV.

### 3.9. Differentiation of SMCs on Different Surfaces

The SMCs on different surfaces were studied for the expression of heavy chain myosin (MYH) and calponin (CAL). MYH expression has never been detected in non-SMCs and is the only marker protein that is also SMC-specific during embryogenesis. CAL is a thin filament protein involved in the regulation of actin-myosin interactions in SMCs.

First, western blotting was performed to identify SMC-specific proteins, MYH and CAL, and to semi-quantify their expression. Briefly, cells on surfaces after 7, 14 and 21 days in culture were homogenized in RIPA lysis buffer (10.0 mM Tris pH 7.4, 100.0 mM NaCl, 5.0 mM EDTA, 5.0 mM EGTA, 1.0% deoxycholate, 0.1% SDS, 1.0% Triton X-100) containing protease inhibitor cocktail. The lysate protein content was determined by a micro-BCA assay. The lysate was heated to 95 °C for 20 min in sample buffer (62.5 mM Tris-HCl pH 6.8, 10.0% glycerol, 5.0% β-mercaptoethanol, 2.0% SDS, 0.025% bromophenol blue) in order to denature the proteins prior to gel loading. An equivalent amount of total extract protein was electrophoresed through 8% Tris-SDS gels and transferred to PVDF membranes in 7.5% methanol. Blots were blocked for 1 h at room temperature. Primary monoclonal antibodies for MYH and CAL were diluted 1:200 in 3% BSA in PBS-tween solution and incubated overnight at 4 °C. The blots were then washed with PBS-tween solution (3 × 5 min) before they were incubated with goat anti-mouse or donkey anti-rabbit horseradish peroxidase (HRP) conjugated secondary antibodies (Santa Cruz Biotechnology) at a dilution of 1:5000 for 1 h at room temperature. The blots were then washed with PBS-tween solution (3 × 5 min) followed by protein detection using chemiluminescence (WestPico Chemiluminescent Substrate; Pierce). The blots were imaged using an Alpha Innotech Fluorchem gel documentation system, and band intensities were analyzed using ImageJ software.

After 21 days of culture, indirect immunofluorescence staining was used to determine the cellular phenotype through the presence of endogenous proteins specific to SMCs when in a mature state. The unadhered SMCs were removed by aspirating the cell-rich media from the surfaces followed by two gentle rinses with PBS. The surfaces were then transferred to a new 48-well plate. Adherent cells were fixed in 3.7 wt% formaldehyde in PBS for 15 min at room temperature and washed in PBS (3 × 5 min). The cell membranes were permeabilized using 1% Triton-X in PBS at room temperature for 3 min and washed in PBS (3 × 5 min). Subsequently, surfaces were incubated in 10% BSA in PBS for 30 min at room temperature. A primary antibody (dilution 1:50, Santa Cruz Biotechnology) with 2% blocking serum in PBS was administered for 1 h at room temperature.

Surfaces were then washed in PBS (3 × 5 min) and incubated with an appropriate secondary fluorescently labeled antibody (dilution 1:100, Santa Cruz Biotechnology, Santa Cruz, CA, USA) with 2% blocking serum in PBS for 1 h at room temperature. The surfaces were washed in PBS (3 × 5 min) and imaged with a fluorescent microscope. All images were processed using ImageJ software. Cellular differentiation and expression were determined by increased fluorescence.

### 3.10. Statistics

Each experiment was confirmed on three different substrates with at least three different cell populations (*n*_min_ = 9). All the quantitative results were analyzed using ANOVA and Tukey’s *post hoc* test. Statistical significance was considered at *p* < 0.05.

## 4. Conclusions

Inhibition of unnecessary vascular SMC proliferation and preservation of a differentiated state in SMCs are important aspects in the management and avoidance of vascular diseases. The level of cell functionality on biomaterial surfaces is also correlated to the characteristics of the surface and its ability to mimic properties similar to that of extracellular matrix. Therefore, providing SMCs with nanotopography and cell-binding motifs, such as collagen, may affect the cell adhesion, viability, morphology and differentiation. In this study, SMCs exhibited increased adhesion on NW, colPCL and colNW surfaces; however, SMCs on nanostructured surfaces seemed to be more elongated than those on PCL surfaces. SEM results also revealed considerable amounts of filopodia interacting with surfaces and neighboring cells on the NW, colPCL and colNW surfaces, but this interaction is lacking on the PCL surfaces. The reduction of MTT was higher on flat surfaces (PCL and colPCL), indicating a higher rate of proliferation. This suggests that SMCs on nanostructured surfaces (NW and colNW) may be in a more differentiated state and slowly dividing. This was confirmed by a significant increase in differentiation markers (CAL and MYH) on these surfaces after seven days in culture without providing cells with differentiation cues. After giving the cells differentiation media, SMCs on all surfaces become spindle shaped. However, the expression of endogenous proteins, CAL and MYH, specific to a contractile SMC phenotype, is upregulated on collagen immobilized surfaces (colPCL and colNW). These results suggest that nanotopography affects cell proliferation, as well as cell elongation, while collagen immobilized surfaces greatly affect cell differentiation with proper differentiation cues.
